# Insights into the development of insulin resistance: Unraveling the interaction of physical inactivity, lipid metabolism and mitochondrial biology

**DOI:** 10.3389/fphys.2023.1151389

**Published:** 2023-04-20

**Authors:** Rachel M. Handy, Graham P. Holloway

**Affiliations:** Department of Human Health and Nutritional Science, University of Guelph, Guelph, ON, Canada

**Keywords:** insulin resistance, metabolism, mitochondria, bioenergetics, skeletal muscle

## Abstract

While impairments in peripheral tissue insulin signalling have a well-characterized role in the development of insulin resistance and type 2 diabetes (T2D), the specific mechanisms that contribute to these impairments remain debatable. Nonetheless, a prominent hypothesis implicates the presence of a high-lipid environment, resulting in both reactive lipid accumulation and increased mitochondrial reactive oxygen species (ROS) production in the induction of peripheral tissue insulin resistance. While the etiology of insulin resistance in a high lipid environment is rapid and well documented, physical inactivity promotes insulin resistance in the absence of redox stress/lipid-mediated mechanisms, suggesting alternative mechanisms-of-action. One possible mechanism is a reduction in protein synthesis and the resultant decrease in key metabolic proteins, including canonical insulin signaling and mitochondrial proteins. While reductions in mitochondrial content associated with physical inactivity are not required for the induction of insulin resistance, this could predispose individuals to the detrimental effects of a high-lipid environment. Conversely, exercise-training induced mitochondrial biogenesis has been implicated in the protective effects of exercise. Given mitochondrial biology may represent a point of convergence linking impaired insulin sensitivity in both scenarios of chronic overfeeding and physical inactivity, this review aims to describe the interaction between mitochondrial biology, physical (in)activity and lipid metabolism within the context of insulin signalling.

## Introduction

**FIGURE 1:**
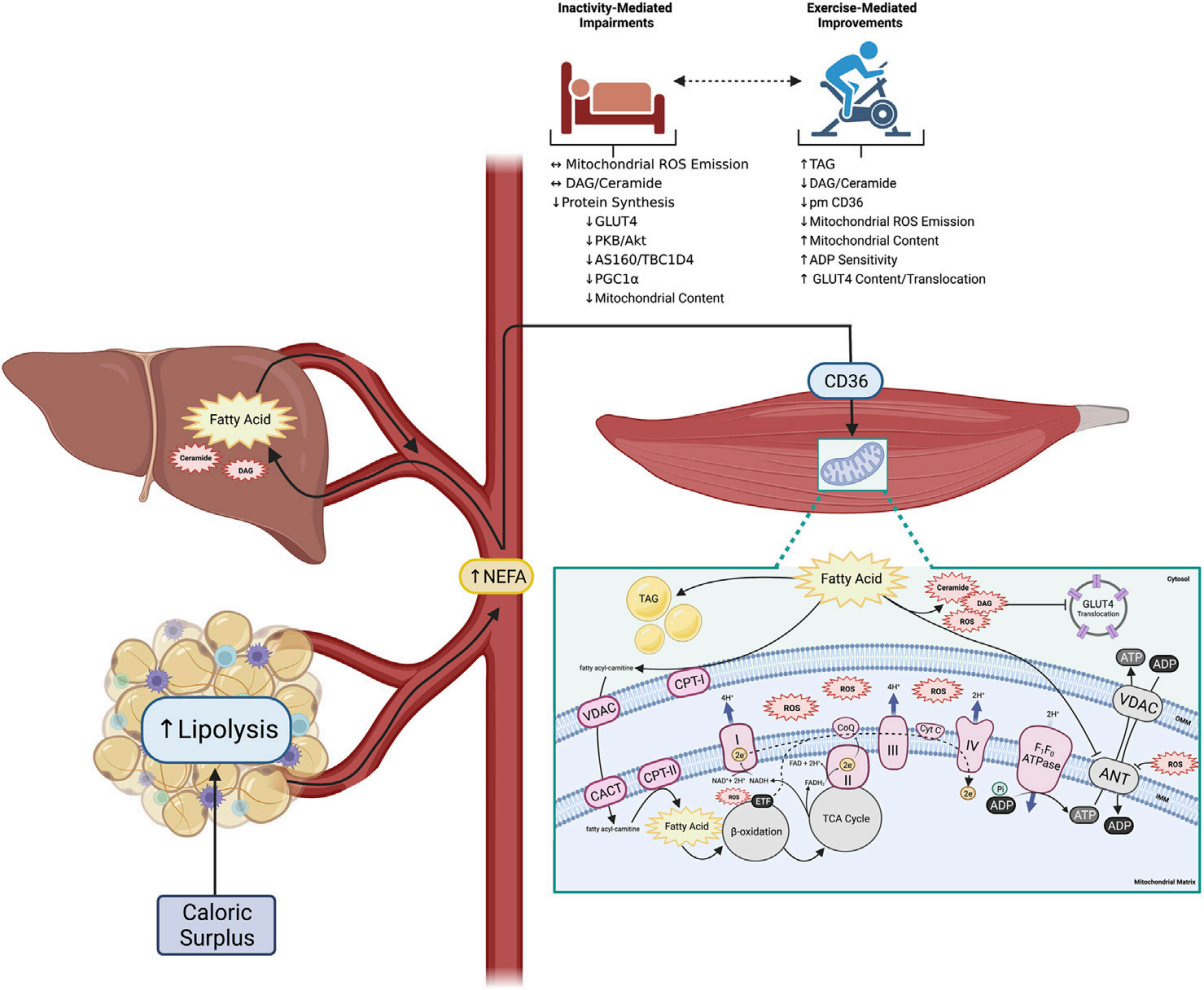
Altered peripheral tissue lipid metabolism initiates skeletal muscle reactive lipid accretion and ROS emission, a mechanism linked to peripheral insulin resistance. Obesity is associated with increased lipid availability (from both an impaired ability to regulate adipose tissue lipolysis, and increased hepatic *de novo* lipogenesis) resulting in increased flux of fatty acids through plasma membrane CD36 in skeletal muscle and the accumulation of various fatty acid species. While some excess fatty acids are converted to TAG for neutral storage, others support the accumulation of reactive lipids (DAG and ceramide). Accumulation of reactive lipids potentiate mitochondrial ROS emission due to increased CPT-I dependent fatty acid transport into the mitochondria, which directly attenuates ADP transport at the level of ANT. Importantly physical inactivity can exacerbate the induction of peripheral insulin resistance, while physical activity can mediate many of the outlined perturbations (created with BioRender.com). Abbreviations: Adenine nucleotide translocase (ANT), adenosine diphosphate (ADP), adenosine triphosphate (ATP), carnitineacylcarnitine translocase (CACT), carnitine palmitoyltransferase-I, -II (CPT-I, -II), cytochrome C (Cyt C), diacylglycerol (DAG), electron (e−), electron transport chain complexes I—IV (I—IV), electron transfer flavoprotein (ETF), fatty acid translocase (CD36), flavin adenine dinucleotide (FADH2), glucose transporter 4 (GLUT4), inner mitochondrial membrane (IMM), nicotinamide adenine dinucleotide (NADH), non-esterified fatty acid (NEFA), outer mitochondrial membrane (OMM), reactive oxygen species (ROS), triacylglycerol (TAG), tricarboxylic acid cycle (TCA), voltage-dependent anion channel (VDAC).

Type 2 diabetes (T2D) remains one of the most significant medical challenges of the 21st century. While the earliest reports of the disease extend back centuries, modern day technical revolutions have resulted in low physical activity combined with the overconsumption of inexpensive, calorically dense, and inadequately satiating food, leading to extraordinary increases in obesity and the incidence of metabolic disorders (Wilmot et al., 2012; Boden et al., 2015). Indeed, in 1995 it was estimated that 135 million adults worldwide had diabetes, a population that was projected to double by 2025 (King et al., 1998). However, more recent reports estimate that 537 million adults were living with diabetes in 2021, and predict a further rise to 783 million by the year 2045 (IDF Diabetes Atlas, 2023), highlighting the disproportional increase in the incidence of metabolic disorders over recent years. While the specific underlying mechanisms that contribute to the development of T2D remain largely ambiguous, it is broadly accepted that excessive caloric intake, especially dietary fat, negatively affects insulin action in key tissues, including white adipose tissue (WAT), liver and muscle (Figure 1). The accumulation of lipids in peripheral tissues contributes to the induction of insulin resistance, defined as an inability of insulin to adequately promote peripheral glucose disposal, suppress lipolysis, and decrease hepatic glucose output. Importantly, the idea that chronic overfeeding may be involved in the development of peripheral insulin resistance originated in 1963 when Randle and colleagues first suggested that a greater availability of substrates (specifically fatty acids) is fundamental to the impaired insulin sensitivity observed in diabetic animals (Randle et al., 1963). Given that post-prandial regulation of whole-body glucose homeostasis is dominated by skeletal muscle and liver function, accumulation of excess lipids in these tissues is believed to be a primary contributor to impairments in glucose tolerance (Hulver et al., 2003; Loomba et al., 2021). Importantly, the excessive presence of lipids within these tissues has been shown to directly (via diacylglycerol (DAG)/ceramide-induced protein kinase C (PKC) signaling) and indirectly (via lipid-mediated mitochondrial reactive oxygen species (ROS) emission) induce insulin resistance. While chronic overfeeding has a well-established role in the development of insulin resistance, it should be acknowledged that physical inactivity represents an independent risk factor for the development of the disease (Wilmot et al., 2012), and occurs in the absence of cellular redox stress and increased skeletal muscle lipid accumulation (Dirks et al., 2016; 2020). As a result, the downstream effects of chronic overfeeding and physical inactivity are likely synergistic with respect to the pathophysiology of insulin resistance in many scenarios.

The regulation of lipid metabolism has been a focus of preventative approaches for decades, and given their primary role in fatty acid oxidation, mitochondria have a considerable impact on the accumulation/metabolism of both neutral (triacylglycerol (TAG)) and reactive (DAG/ceramides) lipids (Koves et al., 2008). In addition to their regulation of substrate metabolism, these organelles also play a significant role in the maintenance of cellular redox homeostasis given their prominent superoxide/ROS production (Brand, 2010). While pharmaceutical interventions that target mitochondrial bioenergetic efficiency have been identified, serious and potentially fatal side effects have rendered many treatment options inadequate (Cutting et al., 1933; Tainter et al., 1933; Cariou et al., 2012). It is now well recognized that exercise is a potent stimulus to induce various insulin sensitising processes, and multiple pathways that produce robust phenotypic changes in the mitochondrial milieu to improve the quantity and quality of the organelle network. Consequently, the inclusion of physical activity to a healthy lifestyle represents an important treatment/prevention strategy for individuals at risk for the development of insulin resistance and T2D.

While several mechanisms have been proposed to regulate mitochondrial quality, including gene transcription, mitophagy and reticulum formation, the importance of these mechanisms are premised fundamentally on affecting mitochondrial bioenergetics. As a result, this review will emphasize the relationship between mitochondrial ‘function’ and insulin signalling before discussing possible mechanisms that negatively influence mitochondrial bioenergetics. Within this framework, we highlight that physical activity has a robust influence on a range of mitochondrial characteristics and can be used as a viable treatment strategy for insulin resistance and T2D.

## Lipid-mediated impairments in peripheral tissue insulin signaling

The accumulation of lipids in non-adipose tissues (namely skeletal muscle and liver) is a distinguished event in various models of insulin resistance (Randle et al., 1963; Marceau et al., 1999; Marchesini et al., 1999; Kim et al., 2000; Ryysy et al., 2000; Boden et al., 2001). Importantly, analysis of the temporal development of whole-body glucose intolerance demonstrate that impairments in skeletal muscle insulin signalling occur secondary to the rapid induction of insulin resistance in WAT following high-fat diet feeding in rodents (Turner et al., 2013). In line with this, impaired suppression of lipolysis in insulin resistant WAT, has been directly linked to increased circulation of non-esterified fatty acids (NEFA) (Boden et al., 1994; Paglialunga et al., 2015), which ultimately supports increased fatty acid uptake into both skeletal muscle (Luiken et al., 2001; Bonen et al., 2004; Han et al., 2007; Holloway et al., 2008) and liver (Westerbacka et al., 2007; Greco et al., 2008; Miquilena-Colina et al., 2011). Importantly, this increase in circulating NEFA appears to be suffucient for the development of whole-body glucose intolerance, evidenced by acute lipid oversupply experiments utilizing intralipid and heparin infusions in humans (Boden et al., 1994; Dresner et al., 1999; Belfort et al., 2005) and rodents (Yu et al., 2002). More recently, reductions in whole-body and hepatic insulin sensitivity following a single dose of palm oil (saturated fat) has been associated with increased rates of hepatic gluconeogenesis and glycogen phosphorylase flux in humans, and differential regulation of hepatic genes involved in canonical insulin signalling in rodents (Hernández et al., 2017). These findings support the classical dogma that increasing lipid supply, directly modulates peripheral tissue insulin signalling and results in the induction of insulin resistance. However, it is likely that the accumulation of neutral lipids (i.e. TAG) is relatively inert. Accordingly, an abundance of evidence demonstrates that the esterification of neutral lipids (i.e., the production of reactive lipid intermediates), in addition to the pathological production of mitochondrial-derived ROS independently contribute to impairments in insulin signalling (Summers, 2006; Liu et al., 2007; Timmers et al., 2008; Anderson et al., 2009; Dubé et al., 2011).

Reactive lipids have been proposed to attenuate insulin signalling through several mechanisms. Within skeletal muscle, DAGs have been demonstrated to activate PKC-θ and PKC-ε in both human and rodent models (Itani et al., 2002; Yu et al., 2002; Szendroedi et al., 2014; Lee et al., 2017). PKC-mediated serine phosphorylation of the insulin receptor substrate (IRS-1) leads to decreased insulin stimulated glucose transport (Yu et al., 2002; Li et al., 2004). The importance of DAG-mediated PKC activation in this pathway has been evidenced with the ablation of PKC-θ in murine skeletal muscle which protects mice from high-fat diet-induced insulin resistance (Kim et al., 2004), as does muscle-specific alanine substitutions on IRS-1 at various serine phosphorylation sites (Morino et al., 2008). In addition to DAGs, the accumulation of ceramides in skeletal muscle may also contribute to insulin resistance by attenuating phosphorylation of protein kinase B (PKB/Akt) (Schmitz-Peiffer et al., 1999; Bruce et al., 2012), a step required for insulin-mediated phosphorylation of Rab-GAP activating proteins (AS160/TBC1D4) and the induction of glucose transporter (GLUT4) translocation to the plasma membrane. Likewise, excessive reactive lipid accumulation within the liver is strongly associated with hepatic insulin resistance. In the liver, DAG-mediated activation of PKC-ε stimulates threonine phosphorylation of the insulin receptor kinase (IRK) at its catalytic subunit (Petersen et al., 2016), which decreases Akt phosphorylation and inhibits insulin signalling. Additionally, impaired insulin-stimulated Akt activity results in decreased insulin-stimulated glycogen synthesis through a reduction of glycogen synthase activity, and decreased insulin inhibition of gluconeogenesis (Samuel et al., 2004; 2007; Petersen et al., 2016). Collectively, DAG inhibits the direct effect of insulin on supressing hepatic glucose production and peripheral tissue glucose disposal, which combined contributes to whole-body glucose intolerance.

The presence of excess lipids and the subsequent production of reactive lipid intermediates is a direct reflection of an imbalance between peripheral tissue lipid supply and intracellular utilization. Given that intracellular lipid utilization occurs via mitochondrial oxidative phosphorylation, an increase in substrate (lipid) delivery/transport to the mitochondrial matrix, beyond what can be readily oxidized, results in the rapid accumulation of reactive lipid intermediates (Itani et al., 2002; Koves et al., 2008) and stimulation of mitochondrial ROS emission (Anderson et al., 2009). While multiple lines of evidence have demonstrated that basal fatty acid oxidation rates (Kelley et al., 1999) and the capacity of skeletal muscle to oxidize lipids (Hulver et al., 2003) are lower in obese/diabetic humans, a compensatory increase in mitochondrial fatty acid oxidation has also been demonstrated at the onset of obesity to buffer the accumulation of reactive lipid intermediates (Helge and Kiens, 1997). From a bioenergetic perspective, an increase in fatty acid oxidation, in the absence of an increase in energy expenditure, increases mitochondrial membrane potential and consequently increases the rate of ROS production (Murphy, 2009; Smith et al., 2021). Further, mitochondrial lipid transport via carnitine palmitoyltransferase-I (CPT-I), which is regarded as the rate limiting step in mitochondrial fatty acid oxidation, is stimulated by substrate provision, as increased lipid concentration has been shown to override the inhibitory effect usually exerted by malonyl-CoA (Smith et al., 2012). Since matrix located enzymes involved in β-oxidation are near-equilibrium, including the electron transfer flavoprotein, which is a potent site of electron leakage to produce ROS (Seifert et al., 2010), combined with the excessive production of NADH, an increase in lipid availability has a high propensity to produce mitochondrial ROS. Importantly, within skeletal muscle this process is unique to lipid provision as carbohydrate oxidation requires flux through the non-equilibrium enzyme pyruvate-dehydrogenase (PDH), which in the absence of allosteric activators/inhibitors of specific phosphatase/kinases is maintained ‘off’ with limited flux (Sugden and Holness, 1994), thus the oxidation of carbohydrates is not controlled by intracellular substrate (i.e. pyruvate) concentration. Essentially at rest, PDH is allosterically inhibited by PDH-kinase (PDK) (Spriet and Heigenhauser, 2002). Given that both PDK content and activity are increased following the acute consumption of a high-fat/low-carbohydrate diet in humans (Peters et al., 2001), and long-term high-fat diet feeding in rodents (Orfali et al., 1993), the ability of carbohydrates to drive mitochondrial ROS production is limited. In support of this, research in murine models indicate that inhibition of reactive lipid synthesis protects against high-fat diet-induced insulin resistance (Ussher et al., 2010; Kurek et al., 2015), while pharmacological (Anderson et al., 2009) and genetic (Lee et al., 2010; Boden et al., 2012) inhibition of mitochondrial ROS emission preserves insulin sensitivity.

While an abundance of evidence suggests that mitochondrial-derived ROS have a primary role in the etiology of insulin resistance, the specific underlying mechanism has yet to be delineated. Nonetheless, it has been proposed that mitochondrial-ROS activate the nuclear factor-κβ pathway (NF-κβ/Iκβ/IKKβ pathway) resulting in serine phosphorylation of IRS-1, and attenuation of insulin signalling in a similar manner to DAGs (Yuan et al., 2001; Sinha et al., 2004). Additionally, both isolated adipocytes and myocytes exposed to H_2_O_2_ demonstrate impaired PKB activation (Tirosh et al., 1999; JeBailey et al., 2007) and markers of insulin-signaling (Rudich et al., 1997; Tirosh et al., 2001; JeBailey et al., 2007), while conversely the use of antioxidants (e.g. lipoic acid or *N*-acetylcysteine) prevent the induction of insulin resistance (Rudich et al., 1999; JeBailey et al., 2007). Though seemingly paradoxical, it should be noted that while chronically elevated mitochondrial H_2_O_2_ emission is detrimental to cellular homeostasis (Anderson et al., 2009), overexpression of the cytosolic H_2_O_2_ antioxidant glutathione peroxidise leads to the development of insulin resistance in mice (McClung et al., 2004). Additionally, neither chronic mitochondrial targeted antioxidant treatment nor enhanced mitochondrial superoxide scavenging prevent HFD-induced insulin resistance despite preserving redox balance (Paglialunga et al., 2012; Lark et al., 2015). Together, these findings indicate that the development of insulin resistance is not exclusively determined by an increase in redox stress, which highlights the complexity surrounding the role of ROS in cellular homeostasis. Indeed, insulin itself has been demonstrated to stimulate the generation of localized ROS, which plays a role in facilitating downstream insulin signalling (Goldstein et al., 2005; Loh et al., 2009; Contreras-Ferrat et al., 2014). In line with this, inhibition of insulin-stimulated H_2_O_2_ production inhibits PI3K activity which corresponds with a ∼50% reduction of insulin-stimulated Akt activation (Mahadev et al., 2001), highlighting the requirement of these molecules for the maintenance of whole-body glucose homeostasis. Collectively, these data emphasize that while low levels of cytosolic ROS are required for the regulation of insulin signalling, in the context of a high-lipid environment (as is observed with chronic caloric overconsumption) both reactive lipid accumulation and mitochondrial ROS production link excessive lipids to many contemporary mechanisms of insulin resistance. However, there remains considerable debate within the scientific community if an intrinsic mitochondrial dysfunction exists, or alternatively that the provision of excess lipid substrates to the mitochondria is inherently problematic as suggested by Randle in 1963.

## Contributions of mitochondrial biology in peripheral tissue insulin resistance

There is an abundance of evidence to suggest that the accumulation of reactive lipids within peripheral tissues can impair various aspects of mitochondrial bioenergetics and is associated with the induction of insulin resistance. Since the first description of mitochondrial dysfunction in the context of glucose intolerance more than 40 years ago (Yamada et al., 1975), mitochondria have been placed as a key organelle in the development of peripheral insulin resistance. While the term ‘mitochondrial dysfunction’ is rather equivocal, it is often used to describe a reduction in the maximal rate of mitochondrial fatty acid oxidation as a result of decreased mitochondrial content and/or activity of key enzymes involved in oxidative phosphorylation. In support of this, an abundance of evidence demonstrates reduced mitochondrial content in insulin resistant/obese individuals (Kelley et al., 2002; Morino et al., 2005; Ritov et al., 2005; Holloway et al., 2007), while genetically increasing mitochondrial content in rodents attenuates lipid-mediated insulin resistance (Koves et al., 2005; Benton et al., 2010; Consitt et al., 2010). In theory, a reduction in mitochondrial fatty acid oxidation would promote accumulation of reactive lipids and increase mitochondrial ROS emission, to ultimately impair peripheral insulin signalling. However, given that obesity is associated with increased plasma free fatty acid concentrations (Randle et al., 1963; Boden, 2003), plasma membrane fatty acid transport (Bonen et al., 2004; 2015), and accumulation of intracellular lipids (Pan et al., 1997; Krssak et al., 2004), the suggestion of reduced mitochondrial fatty acid oxidation cannot be explained by lack of availability and/or tissue delivery. Additionally, the induction of insulin resistance can occur in the absence of reductions in mitochondrial content (Bruce et al., 2005; Holloszy, 2013) or reductions in isolated mitochondrial fatty acid oxidation (Holloway et al., 2007; 2009; Mogensen et al., 2007). These findings in combination with evidence that high-fat diet feeding rapidly increases mitochondrial content alongside the induction of insulin resistance in rodents (Garcia-Roves et al., 2007; Hancock et al., 2008; Miotto et al., 2018), demonstrates that reductions in mitochondrial content are not required in the etiology of insulin resistance. Taken together, there is little evidence to support the hypothesis that an intrinsic impairment in the ability of mitochondria to oxidize fatty acids accounts for increased lipid accretion in peripheral tissues associated with insulin resistance.

While the aforementioned data collectively challenge the relationship between ‘mitochondrial dysfunction’ and insulin resistance, it should be noted that molecular approaches to evaluate mitochondrial function are often performed in the presence of saturating ADP concentrations, which do not reflect the true biological environment. In this regard, it is well-supported that increased mitochondrial biogenesis following aerobic exercise training, results in an enhanced sensitivity of mitochondria to ADP (Holloszy and Coyle, 1984; Dudley et al., 1987), demonstrating that submaximal responses to ADP is likely a key process in regulating cellular homeostasis. Functionally, an increase in mitochondrial ADP sensitivity elicits a reduction in the concentration of ADP required to drive the same absolute respiration (*J*O_2_), which ultimately lowers cytosolic free ADP (Phillips et al., 1996; Perry et al., 2008). While ADP binds to F_1_F_0_ ATPase to reduce membrane potential and ultimately supress mitochondrial ROS emission (Anderson and Neufer, 2006; Anderson et al., 2009), a reduction in ADP transport can attenuate aerobic metabolism and increase mitochondrial ROS emission (Esposito et al., 1999). Interestingly, insulin can enhance submaximal-ADP supported respiration in permeabilized skeletal muscle fibres from both healthy and insulin resistant mice, while the presence of high concentrations of palmitoyl-CoA (P-CoA; to model a high lipid environment) prevented this acute effect (Brunetta et al., 2021). These findings highlight the importance of removing and/or attenuating accumulation of excess lipids to optimize mitochondrial bioenergetics. In line with this, ADP/ATP exchange by adenine nucleotide translocase (ANT) is impaired in the presence of high concentrations of P-CoA, providing proof of principle that lipids can directly impair ADP sensitivity (Ho and Pande, 1974; Ludzki et al., 2015). This concept is supported with the evaluation of submaximal ADP kinetics in various murine models of obesity and T2D, where marked reductions in mitochondrial respiration across a range of submaximal/physiological ADP concentrations is consistently observed (Smith et al., 2013; Miotto et al., 2018). These findings align with what is speculated to occur with the induction of mitochondrial ROS-mediated insulin resistance in a high-lipid environment—whereby the presence of reactive lipids override the inhibition of CPT-I by malonyl-CoA (M-CoA), ‘pushing’ lipids into the mitochondrial matrix, while simultaneously inhibiting ADP transport and submaximal ADP-supported respiration (Smith et al., 2012), both of which would markedly increase mitochondrial ROS emission. However, an avenue of research that has yet to be clearly articulated, is the role of mitophagy in this phenomenon.

Mitochondria are highly dynamic organelles that readily adapt their morphology to the cellular environment—the physiological consequence of overabundant and/or dysfunctional mitochondria is the processes of fission and mitochondrial clearance (mitophagy) to re-establish metabolic homeostasis. While the importance of mitophagy in regulating cellular homeostasis has been previously reviewed (Palikaras et al., 2018), the functional consequence of alterations in mitophagy in the context of insulin resistance is ambiguous. While it may seem logical that reduced mitophagy (i.e., reduced clearance of damaged mitochondrial fragments) may contribute to the enhanced mitochondrial ROS emission and redox stress observed with insulin resistance, recent work has demonstrated contradictory findings. Indeed, high-fat-diet-induced insulin resistance in rodents is associated with increased markers of mitophagy (Ehrlicher et al., 2021), while ablation of the key mitophagy regulating protein FUNDC1 within skeletal muscle does not impair mitochondrial bioenergetics or induce insulin resistance within this tissue (Fu et al., 2018). Thus, it remains unknown whether alterations in mitophagy processes are a cause or consequence of altered mitochondrial bioenergetics. Nonetheless, it could be speculated that the observed increase in mitophagy is a compensatory effort to maintain cellular homeostasis, which may align with alterations in ADP/ATP exchange in insulin resistant tissue (Garcia et al., 2019). Given that ANT is the most abundantly expressed mitochondrial protein (Brand et al., 2005), it is likely susceptible to enhanced redox damage which would account for increased markers of mitophagy alongside alterations in submaximal ADP kinetics observed with chronic obesity/high-fat feeding and prolonged insulin resistance (Brand et al., 2005; Miotto et al., 2018). Taken together, a contemporary model suggests that redox damage on ANT and attenuated ADP/ATP exchange is likely an integral perturbation in the etiology of insulin resistance, caused by the increased availability and transport of fatty acids into the mitochondria.

## Physical inactivity and lipid-independent induction of insulin resistance

Impairments in insulin sensitivity have long been known as a physiological consequence of sedentary behaviour and even short-term inactivity (Smith et al., 2016). This ideology has been evidenced by various step-reduction studies (i.e., <1000–1500 steps/day) which demonstrate negative effects on whole-body glucose homeostasis in as little as 14 days (Knudsen et al., 2012; Breen et al., 2013; McGlory et al., 2018). At the molecular level, this impairment in glucose homeostasis has been associated with a significant decrease in insulin-stimulated skeletal muscle Akt phosphorylation, independent of increased inflammatory markers including TNF and IL-6 (Krogh-Madsen et al., 2010). Further, 7 days of step reduction has been demonstrated to significantly alter the expression of key genes involved in metabolic homeostasis and insulin action (upregulation of SREBP-1C, FAS, GLUT4; downregulation of PDK4, IRS2, HSL) in adipose tissue (Walhin et al., 2013). This is in line with impaired post-prandial glucose control throughout 3 days of reduced (<5,000 steps) physical activity (Mikus et al., 2012). While the specific time-course through which these impairments in insulin action occur remain unknown, a single day of limited physical activity (∼260 steps) has been demonstrated to be sufficient to impair insulin action, evidenced by a ∼39% reduction in the rate of whole-body glucose disappearance in physically active young men and women (Stephens et al., 2011). Importantly, these impairments in glucose homeostasis observed following restriction of physical activity are not rescued within a day of return to normal activity (Reynolds et al., 2015). While the mechanism underlying the induction of insulin resistance following physical inactivity remains unclear, it would appear to be disparate from what is observed following overfeeding.

The exact mechanism(s) causing insulin resistance during acute periods of inactivity remain debatable, as there are inconsistent reports of reactive lipid accumulation (i.e., DAGs and ceramides) (Reidy et al., 2018; Appriou et al., 2019; McKenzie et al., 2020; Kakehi et al., 2021). While substantial increases in DAG concentration have been reported in as little as 3 h of soleus muscle denervation (Turinsky et al., 1990), in humans 7-day bedrest, and 1 day of unilateral limb suspension, induces insulin resistance in the absence of significant changes in skeletal muscle reactive lipid content (Dirks et al., 2016; Bilet et al., 2020). Collectively, these data suggest a dissociation between reactive lipids and the development of insulin resistance within the context of physical inactivity in humans. However, it has been suggested that specific reactive lipids that are not abundant may play an important role in signal transduction (Sowell et al., 1991), which aligns with marginal increases in specific DAG and free fatty acid (FFA) species observed during bedrest-induced insulin resistance (Dirks et al., 2016). Additionally, while this work provides no insight on the subcellular location of lipids, the use of transmission electron microscopy (TEM) has suggested an inverse relationship between lipid accumulation in the SS region and insulin sensitivity in human T2D subjects (Nielsen et al., 2010). Despite the abundance of contradictory findings, it could be speculated that in scenarios of severe physical inactivity, incremental increases in intramyocellular lipid droplets within the SS region influence skeletal muscle insulin signalling, or alternatively that the increase in FFA inhibits mitochondrial ANT/ADP transport and mitochondrial bioenergetics in this specific region of the muscle.

An alternative hypothesis implicates attenuated transcriptional/translational processes and reductions in protein synthesis during the development of skeletal muscle disuse-induced insulin resistance. While an abundance of evidence clearly suggests that a reduction in ambulatory activity results in skeletal muscle atrophy and is often associated with impairments in insulin action and glucose tolerance (reviewed in Oikawa et al., 2019), the underlying mechanism initiating these alterations remain relatively ambiguous. Important to this discussion is the recognition that acute (i.e. 24 h) reductions in ambulatory activity have also been demonstrated to impair glucose homeostasis (Stephens et al., 2011), which would occur independent of significant skeletal muscle atrophy. Thus, impaired synthesis of contractile proteins may not be a plausible explanation for the induction of insulin resistance, at least in an acute model. Alternatively, it could be speculated that attenuated synthesis of proteins involved in canonical insulin signalling and mitochondrial biogenesis have a more significant role in the induction of disuse-mediated insulin resistance over a longer duration of time. Indeed, various studies have associated muscle disuse with a decline in mitochondrial content (determined by citrate synthase activity and oxidative phosphorylation (OXPHOS) protein content) (Alibegovic et al., 2010; Ringholm et al., 2011; Dirks et al., 2016). While rodent models of inactivity are not without limitations (Reidy et al., 2021), these data are supported by reductions in mitochondrial function and content at both the mRNA and protein levels observed following skeletal muscle inactivity (Ljubicic and Hood, 2009a; 2009b; Cartee et al., 2016). Further, bedrest (Biensø et al., 2012; Dirks et al., 2020), and physical inactivity in both humans (Vukovich et al., 1996) and rodents (Host et al., 1998) demonstrate decreased content of key proteins involved in canonical insulin signaling events, including hexokinase (HK), GLUT4 protein, PKB/Akt and AS160/TBC1D4, likely contributing to reduced skeletal muscle glucose transport. In line with this, rapid decreases in expression of peroxisome proliferator-activated gamma coactivator 1-α (PGC-1α), a transcriptional co-activator responsible for the upregulation of widespread gene programs including those involved in insulin signalling and mitochondrial biogenesis, are observed following muscle inactivity (Betik et al., 2009; Cartee et al., 2016). Given that PGC-1α is also known as the master regulator of mitochondrial biogenesis (Puigserver et al., 1998; Wu et al., 1999; Lin et al., 2002), reductions in this protein align with reduced mitochondrial protein content and ADP sensitivity following disuse (Miotto et al., 2019; Dirks et al., 2020; Zuccarelli et al., 2021). This is in contrast to maintained OXPHOS protein content observed in obese and insulin resistant rodents (Garcia-Roves et al., 2007; Hancock et al., 2008; Miotto et al., 2018) which appears to be caused by mitochondrial ROS-mediated ryanodine receptor calcium leak induced gene transcription (Jain et al., 2014). Further, experiments modelling muscle disuse in rodents through denervation or hind limb suspension have demonstrated a significant reduction in the mitochondrial fusion: fission ratio and fragmentation of the mitochondrial reticulum (Iqbal et al., 2013). More recent work has also demonstrated that SS mitochondria from denervated muscle exhibits greater flux in mitophagy markers than the IMF subfraction (Triolo et al., 2022), suggesting that SS mitochondria are likely more sensitive to disuse (Powers et al., 2012b). Importantly, immobilization in humans appears to decrease respiratory capacity faster than declines in OXPHOS protein content (Miotto et al., 2019), suggesting increased mitophagy as a secondary mechanism to remove damaged mitochondrial proteins.

Mechanistically, it has been widely reported that prolonged skeletal muscle disuse results in the accumulation of oxidatively modified proteins and lipids leading to the initiation of various atrophic pathways (Powers et al., 2012a). While mitochondria have been implicated as a key source of inactivity-mediated ROS production in rodent models (Kavazis et al., 2009; Min et al., 2011; Powers et al., 2011), it should be noted that there is an important distinction between the metabolic rate of rodents and humans which may influence the translatability of rodent data. Indeed, rodents demonstrate a ∼8-fold higher metabolic rate than humans (Hudson and Franklin, 2002; Dutta and Sengupta, 2016), which likely contributes to the greater magnitude of ROS production and skeletal muscle atrophy observed in rodent models. Importantly, this paradigm is premised on a significant increase in muscle protein breakdown (MPB) as opposed to a decrease in muscle protein synthesis (MPS), which may not translate to humans. In support of this, physical inactivity is associated with significant reductions in protein synthesis and lean mass (Dirks et al., 2016; McGlory et al., 2018) in the absence of changes in redox stress or mitochondrial ROS emission in humans (Dirks et al., 2016; 2020; Miotto et al., 2019).

## Acute improvements in insulin sensitivity following exercise and influence of training modality

Within 5 years of the discovery of insulin by Banting and Best in 1921, the first reports of exercise-induced improvements in insulin action were published (Lawrence, 1926). Skeletal muscle responds to contraction/exercise with a rapid insulin-independent increase in glucose uptake (reviewed in Richter and Hargreaves, 2013). Notably, exercise also enhances the sensitivity of skeletal muscle to subsequent insulin stimulation (Devlin et al., 1987; Richter et al., 1989; 1989; Cartee et al., 1989) via increased insulin-stimulated translocation of GLUT4 to the muscle membrane following exercise (Hansen et al., 1998). Additionally, the regulation of skeletal muscle membrane permeability to glucose is a key component of exercise-stimulated improvements in whole-body glucose homeostasis. Various studies have demonstrated that the increase in muscle glucose uptake immediately following exercise is mediated by PI3K-independent translocation of GLUT4 to the plasma membrane (Douen et al., 1990; Lee et al., 1995). Importantly, this mechanism is not dependent on insulin, as muscle-specific insulin receptor knockout mice demonstrate increased 2-deoxy-glucose uptake following an acute exercise bout (Wojtaszewski et al., 1999). Collectively, these data provide evidence for an insulin-independent mechanism in the regulation of whole-body glucose homeostasis immediately following exercise. In the 24–72 h following exercise, insulin-dependent pathways, specifically activation of PI3K (Zhou and Dohm, 1997), are upregulated resulting in improved insulin sensitivity during this time. While the specific mechanism leading to PI3K activation remains unclear, some evidence suggests that this occurs independently of IRS-1 (Howlett et al., 2002). This is supported by human data, which demonstrate that improved insulin sensitivity following acute exercise is not caused by increased activity of proximal signalling molecules in the insulin signaling cascade, as downstream kinases appear to be activated first (Wojtaszewski et al., 2000). In line with this, acute exercise has been reported to alleviate lipid-induced insulin resistance in human skeletal muscle by increasing interaction at the level of TBC1D4 (Pehmøller et al., 2012). While the specific mechanism through which TBC1D4 phosphorylation occurs in this scenario is not clear, an insulin-independent mechanism could be speculated given that TBC1D4 contains a phosphorylation domain for both Akt and AMPK. Interestingly, TBC1D4 phosphorylation is maintained for several hours after cessation of exercise, and is further potentiated with insulin stimulation (Sjøberg et al., 2017; Hingst et al., 2018). Additionally, as originally proposed by Holloszy’s group in 2006, the insulin sensitizing effects of exercise are also a function of GLUT4 relocation from the sarcolemma and T-tubules, towards cellular locations that are more accessible for recruitment following insulin stimulation (Geiger et al., 2006). Collectively, these studies demonstrate that acute exercise contributes to whole-body glucose homeostasis via increased glucose transport and transient improvements in insulin sensitivity—both of which provide evidence for acute exercise as a viable therapeutic for insulin resistant and T2D patients, as insulin sensitivity following exercise is conserved in these individuals (Devlin et al., 1987).

Despite clear evidence for the improvement in insulin sensitivity and glycaemic control following exercise, intensity and duration of physical activity may elicit a differential response on peripheral insulin sensitivity. The most consistent evidence for exercise-induced improvements in insulin sensitivity stem from the study of moderate intensity aerobic exercise. Various reports describe improvements in insulin resistance (measured via HOMA-IR), fasting plasma insulin and fasting glucose following 25–60 min of walking or running at 50%–60% VO_2 MAX_ (i.e., low to moderate intensity) three times per week (Damirchi et al., 2014; Herzig et al., 2014; Motahari-Tabari et al., 2014). Insulin sensitivity has also been demonstrated to have a dose-dependent response to aerobic exercise duration, whereby longer exercise duration (i.e., >1900 kcal/week) is associated with further increases in insulin sensitivity and β-cell function in prediabetic individuals (Malin et al., 2013). While longer duration exercise appears to be desirable, the additional improvements in insulin sensitivity when comparing 300 v. 600 kcal/day are marginal (Reichkendler et al., 2014), which is likely an important consideration given that compliance to exercise programs in this population is often low (Ford and Herman, 1995; Troiano et al., 2008; Colley et al., 2011). Notably, the improvements in insulin sensitivity observed with exercise training were diminished within 6 weeks of detraining (Damirchi et al., 2014), indicating the importance of repeated exercise in this response.

The variable of exercise intensity, and the use of high intensity interval training (HIIT) and/or sprint interval training (SIT) in the treatment of metabolic disorders, has gained significant popularity in recent years. HIIT leverages repeated, brief bouts of vigorous exercise separated by short recovery periods, to elicit the metabolic effect of moderate intensity aerobic exercise in a condensed duration. An abundance of literature suggests that HIIT elicits a greater improvement in VO_2_ max (a parameter linked to all-cause mortality) and transient glycaemic control when compared with moderate-intensity, longer duration exercise (Earnest et al., 2013; Racil et al., 2013; Mann et al., 2014; Mitranun et al., 2014). However there remains considerable debate regarding the efficacy of this training program given that a majority of research in this area quantifies acute metabolic responses to HIIT compared with volume-matched lower-intensity endurance exercise, which out of necessity is short in duration and less frequent. However, a recent report utilizing a single-leg, 4-week training program to compare endurance and HIIT adaptations within an individual suggests endurance training elicits greater mitochondrial biogenesis (Skelly et al., 2023), and when not volume matched, endurance exercise also appears to be favorable for improvements in VO_2_ max and post-prandial lipid tolerance when compared to SIT (Petrick et al., 2021). This is in line with the earlier finding that a single bout of endurance exercise prevents impairments in insulin sensitivity following an 18 h lipid infusion (Schenk et al., 2005). Prevention of lipid-induced insulin resistance after endurance exercise has been demonstrated to be accompanied by enhanced skeletal muscle protein expression of key lipogenic enzymes and increases in muscle TAG synthesis (Schenk and Horowitz, 2007), as well as a reduction in the key plasma membrane transporter CD36 abundance in rodents (Smith et al., 2007). Importantly, the partitioning of more fatty acids towards TAG synthesis within muscle following exercise is associated with reduced accumulation of reactive lipid intermediates and decreased phosphorylation/activation of proinflammatory signalling pathways (Schenk and Horowitz, 2007). Conversely, it has been suggested that daily adherence to HIIT elicits an overtraining response and impairs glucose tolerance in trained individuals (Flockhart et al., 2021). However, timing of HIIT exercise may be an important consideration, given evidence to suggest that morning HIIT has an acute, deleterious effect on blood glucose homeostasis, while afternoon HIIT appears to be more efficacious at improving blood glucose in men with T2D (Savikj et al., 2019). While speculative, these observations are likely a function of increases in lipid oxidation occurring in the recovery period as opposed to during the high-intensity exercise bout (as is observed with endurance training). Given that circulating lipids increase overnight (during an approximate 8 h fasting period), it may be logical that this post-exercise increase in lipid oxidation is advantageous. Alternatively, HIIT has been demonstrated to elevate circulating epinephrine (Talanian et al., 2007), which may be problematic in the morning when plasma epinephrine concentrations are naturally elevated (Dodt et al., 1997), resulting in increased hepatic glucose output, thus attenuating the potential benefits of acute exercise on glucose tolerance. Altogether, given the clear importance of daily exercise and the opportunistic timing required for the metabolic benefits of HIIT, these data appear to support endurance exercise as a superior aerobic training modality with respect to regulation of whole-body glucose homeostasis.

While much of the early research on exercise-mediated regulation of insulin sensitivity focused on aerobic exercise training, the use of resistance exercise as a training modality to improve insulin sensitivity should not be overlooked. Primary outcomes in studies evaluating the effects of resistance training in insulin resistant individuals have demonstrated a 10%–15% improvement in insulin sensitivity (Gordon et al., 2009). Similar to aerobic exercise, resistance training increases energy expenditure, which is important for excess substrate oxidation, however, unique to this training modality is skeletal muscle hypertrophy (Maughan et al., 1983), which alleviates reactive lipid accumulation and the induction of insulin resistance (Haines et al., 2020). Importantly this increase in muscle mass is not associated with analogous increases in mitochondrial content (MacDougall et al., 1979), which further divorces the relationship between absolute mitochondrial content and insulin resistance, as mentioned previously. An additional consideration is a combination of moderate intensity aerobic exercise with resistance training which has been demonstrated to significantly increase insulin-mediated glucose uptake compared to individuals performing aerobic exercise alone (Cuff et al., 2003). These findings suggest that combined training for patients with T2D may augment insulin sensitivity via differing mechanisms and importantly, that combined training protocols may elicit additive benefits, however, this could simply be related to a greater volume of physical activity, once again highlighting the importance of regular exercise for metabolic health.

## Molecular mechanisms for chronic exercise-induced changes in insulin sensitivity

While a single bout of exercise causes a transient improvement in insulin sensitivity as outlined above, the molecular mechanisms accounting for improvements in insulin sensitivity induced by chronic exercise training are complex and incomplete. Nonetheless, it is generally accepted that repeated bouts of regular exercise upregulate PGC-1α (Russell et al., 2003; Wright et al., 2007; Perry et al., 2010). While it is well known that PGC-1α upregulates an abundance of genetic programs, including those involved in the canonical insulin signalling cascade (Chibalin et al., 2000; Ryder et al., 2001), it may also be central in the regulation of lipid metabolism. Notably, PGC-1α abundance is positively correlated with enhanced fatty acid oxidation (Huang et al., 2017), which associates training induced improvements in peripheral lipid handling. Specifically, co-activation of peroxisome proliferator-activated receptor (PPAR) by PGC-1α upregulates the TAG synthesizing enzymes DAG acyltransferase 1 (DGAT1) (Summermatter et al., 2010); and perilipin (Arimura et al., 2004), both of which promote lipid storage as neutral TAG. This is evidenced by enhanced TAG synthesis and reduced DAG and ceramide accumulation in skeletal muscle following adherence to chronic training in humans and rodents (Goodpaster et al., 2001; Holloway et al., 2014). However, the consideration of subcellular location of intramuscular lipid droplets is important, given the relationship between lipid accumulation in the SS region and insulin resistance in both humans and rodents (Nielsen et al., 2010; Lally et al., 2012). Importantly, intramyocellular lipid droplets stored in the SS region are used to a greater extent during exercise (Stellingwerff et al., 2007), a finding that extends to T2D subjects who demonstrate decreased SS lipids following aerobic exercise training (Nielsen et al., 2010). Despite the clear metabolic improvements observed with chronic exercise training, repeated exercise is paramount to these responses. In support of this concept, many models demonstrate that the absence of training for 3 to 5 days is sufficient to mitigate the benefits of chronic training (Roberts et al., 2013). This aligns with original research in this area which demonstrates a 50% reduction in the activities of key mitochondrial enzymes, citrate synthase and succinate dehydrogenase, 12 days following the cessation of prolonged intense endurance training (Coyle et al., 1984). As such, an evolving area of research has focused on the induction of mitophagy within skeletal muscle in response to exercise and detraining.

While general apoptosis is not linked to exercise (Quadrilatero et al., 2010), our understanding of the potential role of mitophagy in response to exercise is still emerging. Recent work suggests that the energy sensor AMPK is required for the increase in mitophagy that is observed 6 hours following acute treadmill exercise (Laker et al., 2017) and elicits an increase in Parkin localization to muscle mitochondria (Vainshtein et al., 2015; Chen et al., 2018). Importantly, an increase in mitochondrial Parkin localization following acute treadmill exercise, aligns with an increase in mitophagy flux (measured via LC3-II, p62, and ubiquitin) which remains elevated post-exercise (Vainshtein et al., 2015; Chen et al., 2018). Further, the observed increase in mitophagy flux were not present in Parkin-knockout animals (Chen et al., 2018), suggesting that Parkin is required for the mitophagy response to endurance exercise. While these studies have focused on an acute response to exercise, other research has demonstrated that basal mitophagy is elevated in response to 5 weeks of swimming (Dun et al., 2017) or 4 weeks of voluntary wheel running (Lira et al., 2013), evidenced by an increased LC3-II:LC3-I ratio, decreased p62, and elevated BNIP following both interventions. Additionally, endurance trained mice have elevated Parkin protein expression and mitochondrial localization, as well as a blunted mitophagy response to acute exercise (Chen et al., 2018), while studies in humans have demonstrated that endurance training results in elevated phosphorylation of Pink1 and Parkin (Tarpey et al., 2017), both of which suggest enhanced specificity of mitophagy in endurance trained muscle. Given that classical training adaptations confer increases in mitochondrial content, an improved sensitivity of various enzymes involved in metabolic signalling (i.e., AMPK, p38, CaMK) would be expected (Dudley et al., 1987; Ljubicic and Hood, 2009b) and aligns with reductions in mitophagy following acute physical activity in endurance trained individuals (Chen et al., 2018). While speculative, it is likely that these alterations to mitophagy flux initiate remodelling of the existing mitochondrial milieu, which may be one mechanism through which exercise modulates mitochondrial dynamics and improves peripheral insulin sensitivity.

## Conclusion

In this review, we aimed to highlight the central role of mitochondrial biology in various mechanisms thought to underly the etiology of peripheral insulin resistance. The accumulation of lipids in non-adipose tissues is a distinguished event in various models of insulin resistance, resulting in both direct (via reactive lipids) and indirect (via mitochondrial ROS emission) impairments in peripheral insulin signalling. While several mechanisms have been proposed to regulate mitochondrial quality, including gene transcription, mitophagy and reticulum formation, the importance of these mechanisms are fundamentally premised on affecting mitochondrial biogenesis. However, clear evidence for the presence of impaired mitochondrial oxidative capacity in the induction of insulin resistance does not exist, and redox stress and reactive lipid accretion appears to be specific to scenarios of chronic overfeeding. Alternatively, it appears that lipids can competitively and/or allosterically inhibit mitochondrial ADP transport as a key event negatively affecting mitochondrial bioenergetics. In contrast to a high lipid environment, extreme physical inactivity attenuates protein synthesis and reduces mitochondrial and canonical insulin signaling proteins as alternative mechanisms-of-action to limit insulin action. While the effects of lipid-induced metabolic perturbations and physical inactivity are therefore likely additive with respect to the pathophysiology of insulin resistance, since exercise stimulates robust phenotypic changes in lipid homeostasis, mitochondrial bioenergetics and protein synthesis/gene transcription, the inclusion of physical activity represents an important treatment/prevention strategy for individuals at risk of the development of insulin resistance and T2D.
